# Nutritional Programming of Bone Structure in Male Offspring by Maternal Consumption of Citrus Flavanones

**DOI:** 10.1007/s00223-017-0366-0

**Published:** 2017-11-18

**Authors:** Sandra M. Sacco, Caitlin Saint, Paul J. LeBlanc, Wendy E. Ward

**Affiliations:** 1 Department of Kinesiology, Faculty of Applied Health Sciences, Brock University, 1812 Sir Isaac Brock Way, St. Catharines, ON L2S 3A1 Canada; 2 Centre for Bone and Muscle Health, Brock University, St. Catharines, ON L2S 3A1 Canada; 3 Department of Health Sciences, Faculty of Applied Health Sciences, Brock University, St. Catharines, ON L2S 3A1 Canada

**Keywords:** Bone structure, Hesperidin, In vivo micro-computed tomography, Mice, Naringin, Nutritional programming

## Abstract

Maternal exposure to hesperidin (HSP) and naringin (NAR) during pregnancy and lactation transiently compromised bone mineral density (BMD) and bone structure at the proximal tibia in female CD-1 offspring. We examined whether maternal consumption of HSP + NAR during pregnancy and lactation compromises BMD, bone structure, and bone strength in male CD-1 offspring. Male CD-1 offspring, from mothers fed a control diet (CON, *n* = 10) or a 0.5% HSP + 0.25% NAR diet (HSP + NAR, *n* = 8) for 5 weeks before mating and throughout pregnancy and lactation, were weaned and fed CON until 6 months of age. In vivo micro-computed tomography (µCT) measured tibia BMD and structure at 2, 4, and 6 months of age. Ex vivo µCT measured femur and lumbar vertebrae (LV) structure at age 6 months. Ex vivo BMD (femur, LV) and biomechanical strength (femur and tibia midpoint, femur neck) were assessed at age 6 months by dual energy x-ray absorptiometry and strength testing, respectively. At all ages, HSP + NAR offspring had greater (*p* < 0.05) proximal tibia cortical structure compared to CON offspring. At age 4 months, proximal tibia trabecular structure was greater (*p* < 0.05) than CON offspring. At age 6 months, femur neck and LV trabecular structure were greater (*p* < 0.05) than CON offspring. Our results demonstrate that unlike our previous study of female offspring, maternal consumption of HSP + NAR resulted in greater bone structure at the proximal tibia in male CD-1 offspring that persisted to 6 months of age. Thus, maternal programming of offspring BMD and bone structure from consumption of HSP + NAR occurred as a sex-specific response.

## Introduction

Nutritional programming is the phenomenon that occurs when the presence or the level of a specific food or food component consumed during pregnancy and/or the early neonatal period exerts long-lasting effects on the phenotype of the offspring. Epigenetic mechanisms may be responsible for these long-lasting effects by causing modifications to gene expression without altering the nucleotide sequence itself (reviewed in [1, 2]). There is increasing evidence in various animal models showing that bone health can be programmed by changes in nutrition that occur in utero and/or during the neonatal period. Maternal or early life exposure to a high-fat diet [3], protein undernutrition [4], as well as supplementation with micronutrients including folic acid [5] and vitamin D [6] has been shown to favorably [5, 6] or unfavorably [4] program bone health in growing and adult offspring. In addition to these data, our laboratory has shown that bioactives such as citrus flavanones [7] and soy isoflavones [8–10] set a trajectory for better [8–10] or compromised [7] bone health in adult CD-1 offspring and that effects in offspring are sometimes sex-specific.

Hesperidin (HSP) and naringin (NAR) are flavanone glycosides belonging to the class of flavonoids that are found abundantly in citrus fruits and juices [11, 12]. HSP is present at exceptionally high levels in orange peel and is formed from the flavanone hesperetin and rutinose disaccharide. The content of NAR is particularly high in grapefruit and consists of the flavanone naringenin bound to the disaccharide neohesperidose [13]. In male orchidectomized or senescent rats, the consumption of HSP, NAR, or their combination protected against the loss of BMD and biomechanical bone strength and deterioration of bone structure [14–16], effects that were attributed to decreases in osteoclast number [15] and an attenuation in bone resorption [14, 16]. Thus, HSP and NAR support bone metabolism to preserve bone health during aging and these actions are attributed to their aglycone and glucuronide metabolites that are formed during enzymatic digestion in the intestinal tract and glucuronidation in the liver [11, 14, 17–19].

Findings from in vitro studies suggest a potential role for HSP and NAR to support bone development. Specifically, HSP and NAR stimulate the expression of key factors that promote osteoblast differentiation [14, 17, 18, 20–22]. Despite these data, no studies have examined whether and how HSP or NAR supports the development of BMD, bone structure, or biomechanical strength in vivo. Recently, we have shown a nutritional programming effect of maternal consumption of citrus flavanones. Female CD-1 offspring from mothers fed a diet containing 0.5% HSP combined with 0.25% NAR before mating and throughout pregnancy and lactation had compromised trabecular bone structure at the proximal tibia at 2 and 4 months of age and lower trabecular BMD at 4 months of age compared to offspring from mothers fed the control diet [7]. These detriments to BMD and bone structure were no longer observed at 6 months of age, indicating that maternal consumption of HSP + NAR exerts transient detriments to bone development in female CD-1 offspring. These findings prompt the question of whether male siblings may experience similar detriments to BMD and bone structure. Sex-specific nutritional programming effects on the skeletal health of rodents have been observed using other food bioactives [3, 6, 10, 23]. Thus, the objective of the present study was to determine if maternal consumption of HSP + NAR during pregnancy and lactation compromises BMD, bone structure, and biomechanical strength in male CD-1 offspring throughout development and into early adulthood.

## Materials and Methods

### Animals and Diets

Eighteen female CD-1 mice (5 weeks old) and 8 male CD-1 mice (8 weeks old) were received from Charles River Canada (St. Constant, QC, Canada). Animals were housed 4–5 mice per cage in a temperature-controlled room (22–24 °C) at 50% humidity, and a 12:12 h light:dark cycle. Female mice were randomly assigned to control diet (AIN-93G, CON, *n* = 10) for growth, pregnancy, and lactation [24, 25] or the CON diet that was supplemented with HSP and NAR (HSP + NAR, *n* = 8). The CON diet (TD. 06706, Harlan Teklad, Mississauga, ON, Canada) was a modified AIN-93G diet that contained alcohol-extracted casein which removed vitamins that are naturally occurring in casein (i.e., folate) and that may exert programming effects that alter bone development [5]. The HSP + NAR diet consisted of 0.5% w/w HSP (H5254, Sigma-Aldrich, Oakville, ON, Canada) and 0.25% w/w NAR (N1376, Sigma-Aldrich, Oakville, ON, Canada) that were added to the CON diet at the expense of cornstarch. Throughout the study, food intake was measured twice per week and all mice had ad libitum access to food and water. All applicable international, national, and institutional guidelines for the care and use of animals were followed and the experimental protocol (AUP 14-04-01, 2014) was approved by the Animal Care Committee at Brock University, St. Catharines, ON, Canada.

### Experimental Design

Female and male mice were mated harem-style at 10 weeks of age during which they were maintained on the respective diets of the female. Female mice were then housed individually once they were identified as pregnant, and were kept on their respective diets until the end of lactation. The present study reports on the male offspring and findings from their female siblings have been reported elsewhere [7]. Litters weights were recorded at post-natal day (PND) 9, PND 16, and PND 21 using an electronic scale. Litter weights were similar between CON and HSP + NAR groups [7]. At PND 21, all male offspring were weaned onto the CON diet until 6 months of age. Mice were housed 4–5 mice per cage and received ad libitum access to the CON diet and water. Food intake was measured twice per week and body weight was measured once per week using an electronic scale. Mean daily food intake for each mouse was estimated by taking the mean food intake per week for each cage and dividing it by number of days and by the number of mice per cage.

#### Structure of Tibias (In Vivo) and Femurs and Lumbar Vertebrae (Ex Vivo)

An in vivo µCT scanning system (SkyScan 1176, Bruker microCT, Kontich, Belgium) was used to assess the bone mineral density (BMD) and bone structure at the right tibia of mouse offspring at 2, 4, and 6 months of age as previously described [7, 26]. Briefly, general anesthesia was induced using 2–5% isoflurane dissolved in 100% oxygen and mice were then transitioned to a nose cone to maintain anesthesia and placed on the scanning bed. The right tibias were then scanned using in vivo µCT scanning as previously described [26]. Scanning parameters included an isotropic voxel size of 9 μm^3^, 1 mm aluminum filter to reduce beam hardening, 40 kV tube voltage, 300 μA amperage, 3350 ms integration time, a rotation step of 0.8° over 180° to achieve acceptable contrast. Frame averaging was not performed during scanning and total scan time was 16 min 23 s. Each scan resulted in exposure of the scanned tibia to ionizing radiation at a dose of 460 mGy per scan (TN-502RD-H, Best Medical Canada, Ottawa, ON, Canada). This dose does not affect tibial bone structure when tibias are repeatedly exposed to 460 mGy from in vivo µCT scanning at 2, 4, and 6 months of age [26]. Five days after the last scan, 6-month-old mice were euthanized by exsanguination under anesthesia (5% isoflurane dissolved in 100% oxygen), followed by cervical dislocation. Skeletal tissues including tibias, femurs, and lumbar vertebrae (LV 1–6) were excised and cleaned from soft tissue before they were wrapped in saline-soaked gauze and stored at − 80 °C.

To minimize exposure to ionizing radiation, the right femurs and second lumbar vertebra (LV2) were scanned ex vivo using µCT scanning (SkyScan 1176, Bruker microCT, Kontich, Belgium) to assess the trabecular bone structure at the femur neck, distal femur, and LV2 and the cortical bone structure and geometry at the femur midpoint and LV2 of male offspring as previously described [7]. The bones were scanned using the following scanning parameters: 9 µm^3^ isotropic voxel size, 0.25 mm aluminum filter, voltage of 45 kV, tube current of 545 µA, 850 ms exposure time, and 0.2° rotation step over 180°. Frame average was not performed and the total scan time was 40 min 54 s for each scan.

#### Post-Scanning Image Processing and Analysis

Cross-section images from the tomography projection images were reconstructed using NRecon Reconstruction 64-bit software (SkyScan, Bruker microCT, Kontich, Belgium) coupled with Graphics Processing Unit (GPU)-acceleration (GPUReconServer, SkyScan, Bruker microCT, Kontich, Belgium) as previously described [7]. Except for variable misalignment compensations, the same reconstruction parameters and corrections were applied across all scanned images at each skeletal site. Reconstructed images were then reoriented using DataViewer software (version 1.5.0, SkyScan, Bruker microCT, Kontich, Belgium), and the transaxial images were saved. Regions of interest (ROIs) for trabecular and cortical bone at the proximal tibia were selected by automatically segmenting trabecular and cortical bone from one another. ROIs for trabecular bone at the femur neck, distal femur, and LV2, and for cortical bone at the femur midpoint and LV2 were manually drawn and saved as new datasets as previously described [7]. For the ROI at the tibia midpoint, a total of 100 transaxial slices (0.880 mm in length) were selected spanning above and below the midpoint and a new dataset was saved. Task lists (CT Analyzer software, SkyScan Bruker microCT, Kontich, Belgium) were then generated and applied to the trabecular bone ROI datasets at the proximal tibia, femur neck, distal femur, and LV2 and to the cortical bone ROI datasets at the proximal and midpoint tibia, midpoint femur, and LV2 to segment bone from the background. Local and global thresholding were used to segment trabecular and cortical bone, respectively, from the background as previously described [7]. At the tibia midpoint, global thresholding was used to segment cortical bone from the background, using a lower threshold of 120 and an upper threshold of 255.

Three-dimensional µCT outcome measures of trabecular bone determined at the proximal tibia, femur neck, distal femur, and LV included bone volume (BV, mm^3^), total volume (TV, mm^3^), bone volume fraction (BV/TV, %), trabecular number (Tb.N, mm^−1^), trabecular thickness (Tb.Th, mm), trabecular separation (Tb.Sp, mm), connectivity density (Conn.D, 1/mm^3^), and degree of anisotropy (DA, no unit). Two-dimensional µCT outcome measures of cortical bone at the proximal and midpoint tibia, and femur midpoint included cortical bone area (Ct.Ar, mm^2^), total cross-sectional area inside the periosteal envelope (Tt.Ar, mm^2^), cortical area fraction (Ct.Ar/Tt.Ar, %), average cortical thickness (Ct.Th, mm), periosteal perimeter (Ps.Pm, mm), endocortical perimeter (Ec.Pm, mm), marrow area (Ma.Ar, mm^2^), and eccentricity (no unit). Two-dimensional µCT outcome measures of cortical bone at LV2 included cortical bone area (Ct.Ar, mm^2^) and average cortical thickness (Ct.Th, mm).

#### Bone Mineral Density (BMD) of Tibias, Femurs, and LV1-3

In vivo µCT scanning (SkyScan 1176, Bruker microCT, Kontich, Belgium) was used to assess trabecular BMD at the proximal tibia and cortical tissue mineral density (TMD) at both the proximal tibia and tibia midpoint at 2, 4, and 6 months of age. 0.25 and 0.75 g/cm^3^ calcium hydroxyapatite calibration phantoms were scanned and processed by applying the parameters selected for the in vivo scans of the tibias. BMD was calibrated against the attenuation coefficient, and trabecular BMD at the proximal tibia and cortical TMD at the proximal and midpoint tibia were subsequently measured against the attenuation coefficient [27]. To determine BMD at the femur and LV1-3, dual energy x-ray absorptiometry (DXA) (Orthometrix, White Plains, NY, USA) with a specialized software program (Host Software version 3.9.4; Scanner Software version 1.2.0, Orthometrix, White Plains, NY, USA) was used as previously described [7].

#### Peak Load of Tibias and Femurs

Three-point bending at the midpoint of the tibia and femur and compression testing at the femur neck were performed using a Materials Testing System (Model 4442, Instron Corp., Norwood, MA, USA) and specialized software (Bluehill 2, Instron Corp., Norwood, MA, USA) as previously described [7, 28].

#### Statistical Analysis

All data are presented as mean ± SEM. To determine the effects of age, diet, and their interaction on food intake, body weight, in vivo trabecular bone structure at the proximal tibia, and in vivo cortical bone structure at the proximal tibia and tibia midpoint, two-way repeated measures ANOVAs (general linear model) were performed. If significant interactions were observed (*p* < 0.05), post hoc simple main effects were examined using Bonferroni’s correction for multiple comparisons. If no significant interactions were observed, main effects were examined to determine whether there were any differences in bone structural outcome measures between CON and HSP + NAR groups at each age or, whether there were any differences over ages within each group. Missing values resulting from leg movement during scans were replaced with the series mean. This occurred once in the CON group at 2 months of age, once in the HSP + NAR group at 2 months of age, and once in the HSP + NAR group at 4 months of age. Comparisons of ex vivo mineral, structure, and strength properties between CON and HSP + NAR groups at 6 months of age were assessed by unpaired, two-tailed Student’s *t* tests. All statistical analysis was conducted using SPSS Statistics (version 23, IBM, Armonk, NY, USA) and *p* < 0.05 was considered statistically significant.

## Results

### Food Intake and Body Weight

**Fig. 1:**
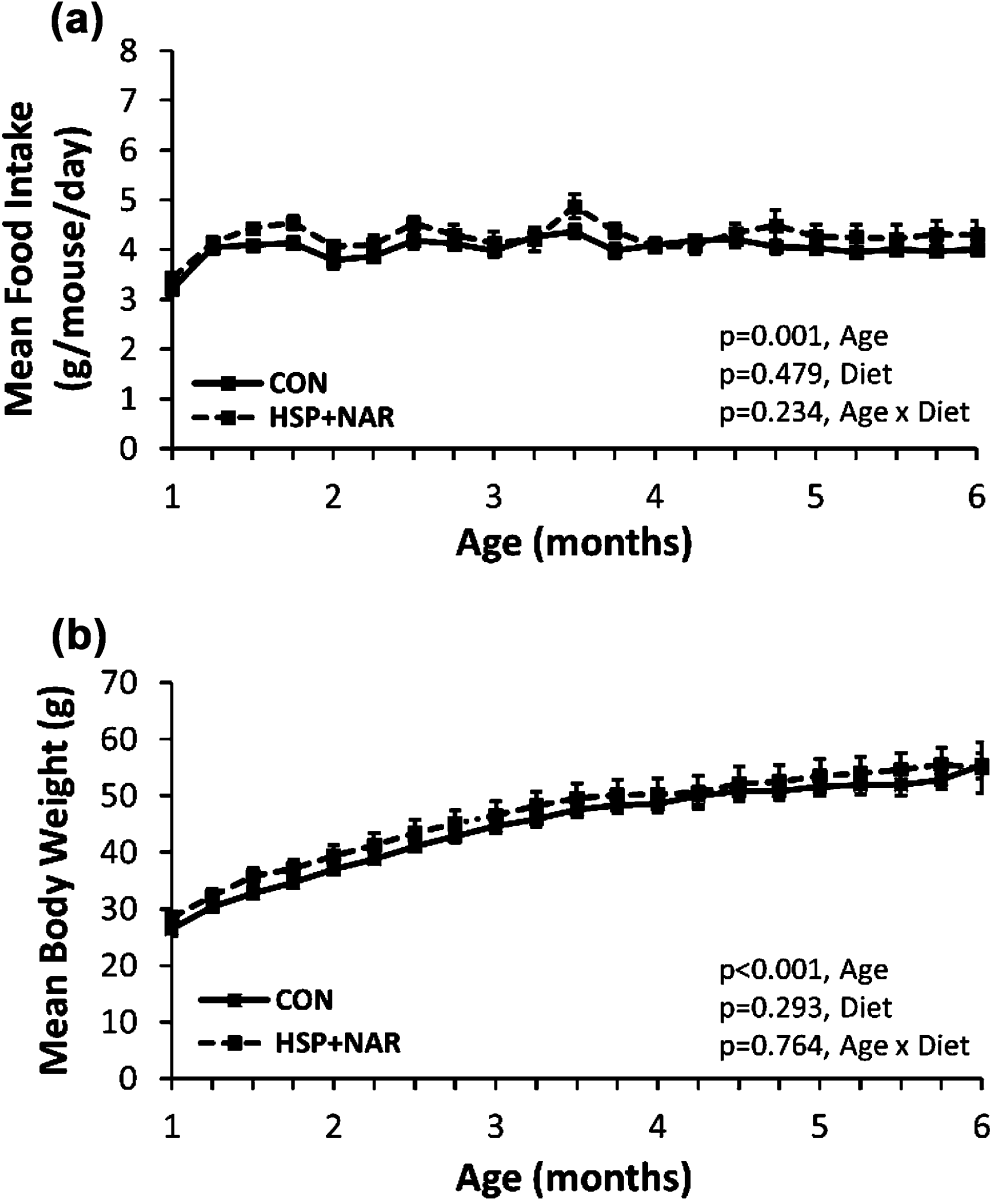
Effect of maternal consumption of 0.5% hesperidin (HSP) combined with 0.25% naringin (NAR) on food intake (**a**) and body weight (**b**) of male CD-1 offspring. Female dams were fed HSP + NAR or the control (CON) diets throughout pregnancy and lactation. Upon weaning, male offspring were fed the CON diet until 6 months of age. Data are expressed as mean ± SEM. *n* = 10 (CON group), *n* = 8 (HSP + NAR group)

From 1 to 6 months of age, a significant main effect of age was observed for mean daily food intake (*p* = 0.001, Fig. 1a) and body weight (*p* < 0.001, Fig. 1b) in male offspring whereby food intake and body weight increased over the study duration. There were no main effects of diet on mean daily food intake (*p* = 0.479) or body weight (*p* = 0.293) nor were there any significant two-way interactions observed between age and diet on food intake (*p* = 0.234) or body weight (*p* = 0.764) (Fig. 1).

### In Vivo Trabecular and Cortical BMD and Structure at the Proximal and Midpoint Tibia

**Fig. 2:**
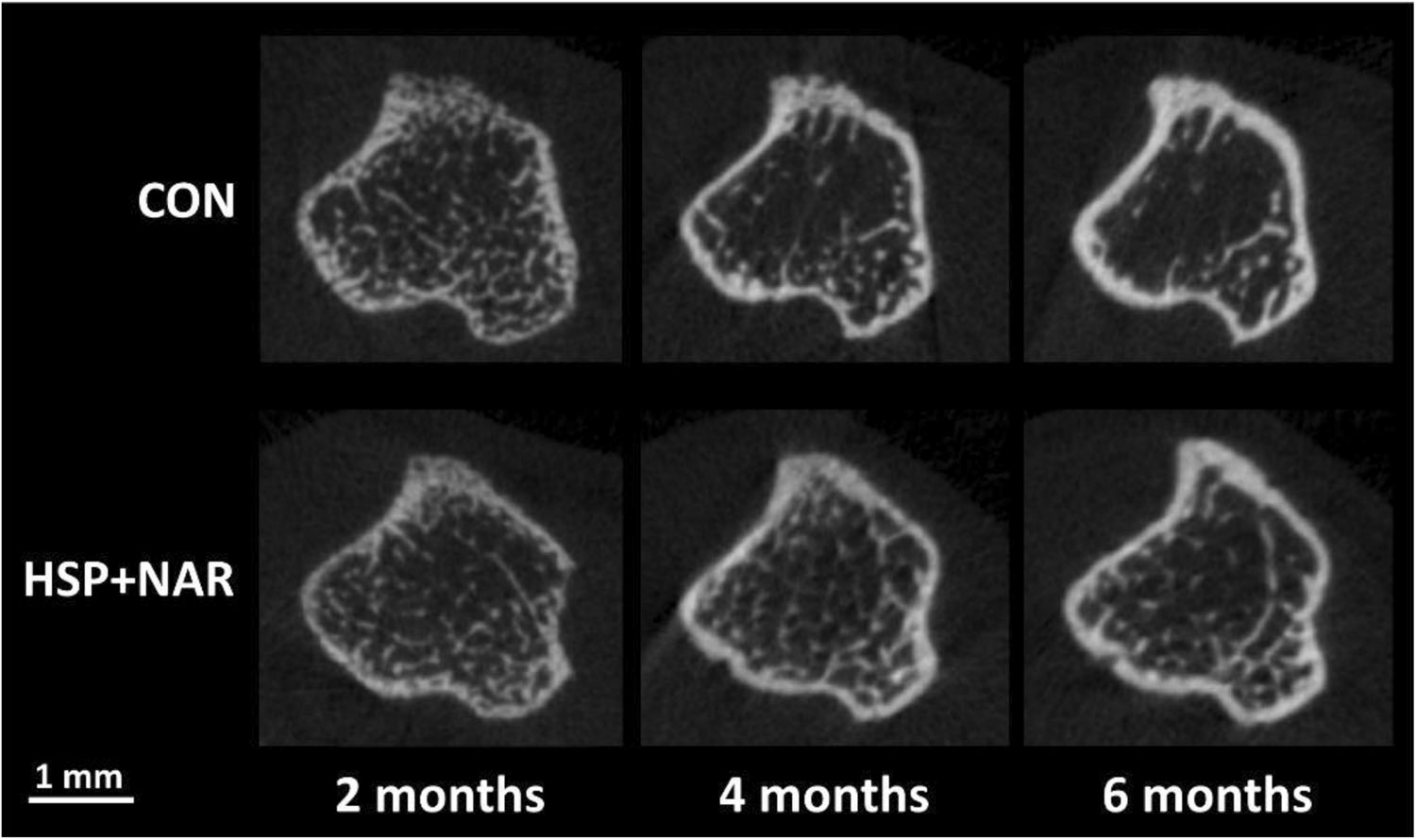
Representative images of trabecular and cortical bone structure at the right proximal tibia of male CD-1 offspring at 2, 4, and 6 months of age from mothers that were fed 0.5% hesperidin (HSP) combined with 0.25% naringin (NAR) or the control (CON) diet throughout pregnancy and lactation. Images within the CON and HSP + NAR groups represent the right proximal tibia scanned in vivo from the same mouse at 2, 4, and 6 months of age

For trabecular bone at the proximal tibia, a significant two-way interaction between age and diet was observed for multiple trabecular outcome measures including TV (*p* = 0.028), BV (*p* = 0.006), BV/TV (*p* = 0.010), and Tb.N (*p* = 0.020) (Table 1). Simple main effects conducted to distinguish differences between CON and HSP + NAR groups over ages demonstrated that BV (*p* = 0.041), BV/TV (*p* = 0.018), and Tb.N (*p* = 0.038) were significantly greater in the HSP + NAR group compared to CON at 4 months of age and not at 2 or 6 months of age (Table 1, Fig. 2). For TV, maternal consumption of HSP + NAR resulted in lower (*p* = 0.006) TV in male offspring compared to CON offspring at 2 months of age but not at 4 or 6 months of age. In addition to the interaction effects between age and diet, a significant main effect for diet was observed for Tb.Th (*p* = 0.033) whereby maternal consumption of HSP + NAR resulted in higher (*p* = 0.016) trabecular thickness at 4 months of age compared to offspring from mothers fed the CON diet. A main effect of age (*p* < 0.01) was observed for trabecular parameters at the proximal tibia whereby TV, BV, and Conn.D decreased and Tb.Th increased from 2 to 6 months of age in both CON and HSP + NAR groups (Table 1, Fig. 2). In addition, main effects of age (*p* = 0.002) were observed for Tb.N and Tb.Sp whereby Tb.N decreased and Tb.Sp increased from 2 to 6 months of age in the HSP + NAR group.

**Table 1:** Trabecular and cortical bone mineral density (BMD) and bone structure of the right proximal tibias of male CD-1 offspring from mothers that were fed a control (CON) or 0.5% hesperidin (HSP) + 0.25% naringin (NAR) diet throughout pregnancy and lactation

Outcomes	CON			HSP + NAR			*p* value		
	Age (months)								
	2	4	6	2	4	6	Age	Diet	Age × diet
Trabecular structure									
BMD (g/cm^3^)	0.200 ± 0.008	0.193 ± 0.006	0.194 ± 0.008	0.216 ± 0.006	0.219 ± 0.011	0.199 ± 0.011	0.082	0.143	0.051
TV (mm^3^)	1.496 ± 0.410^a^	1.163 ± 0.042^b^	1.134 ± 0.049^b^	1.324 ± 0.034*^a^	1.126 ± 0.030^b^	1.020 ± 0.039^c^	*p* < 0.001	0.114	0.028
BV (mm^3^)	0.101 ± 0.014^a^	0.066 ± 0.007^b^	0.066 ± 0.009^ab^	0.100 ± 0.011^ab^	0.098 ± 0.014*^a^	0.064 ± 0.013^b^	0.005	0.485	0.006
BV/TV (%)	6.66 ± 0.770	5.58 ± 0.49	5.73 ± 0.68	7.46 ± 0.67^ab^	8.65 ± 1.15*^a^	6.02 ± 1.09^b^	0.059	0.164	0.010
Tb.Th (mm)	0.057 ± 0.001^c^	0.063 ± 0.001^b^	0.070 ± 0.001^a^	0.059 ± 0.001^b^	0.068 ± 0.001*^a^	0.072 ± 0.002^a^	*p* < 0.001	0.033	0.299
Tb.N (1/mm)	1.151 ± 0.113	0.885 ± 0.077	0.824 ± 0.100	1.264 ± 0.098^a^	1.285 ± 0.173*^a^	0.858 ± 0.165^b^	0.002	0.230	0.020
Tb.Sp (mm)	0.324 ± 0.013	0.359 ± 0.014	0.381 ± 0.016	0.309 ± 0.0149^b^	0.330 ± 0.026^ab^	0.382 ± 0.029^a^	0.002	0.522	0.366
DA (no unit)	2.544 ± 0.103	2.396 ± 0.197	2.196 ± 0.216	2.568 ± 0.154	2.044 ± 0.147	2.518 ± 0.279	0.051	0.993	0.140
Conn.D (1/mm^3^)	46.4 ± 3.7^a^	21.3 ± 2.4^b^	22.8 ± 3.8^b^	44.2 ± 4.89^a^	36.9 ± 6.6*^ab^	24.5 ± 2.4^b^	*p* < 0.001	0.240	0.075
Cortical structure									
TMD (g/cm^3^)	0.716 ± 0.007^c^	0.882 ± 0.008^b^	0.919 ± 0.011^a^	0.749 ± 0.008^c^	0.892 ± 0.011^b^	0.935 ± 0.019^a^	*p* < 0.001	0.098	0.299
Ct.Ar (mm^2^)	1.091 ± 0.053^c^	1.222 ± 0.035^b^	1.323 ± 0.041^a^	1.284 ± 0.051*^b^	1.372 ± 0.063*^b^	1.507 ± 0.059*^a^	*p* < 0.001	0.014	0.633
Tt.Ar (mm^2^)	4.376 ± 0.100^a^	3.748 ± 0.108^b^	3.802 ± 0.121^b^	4.179 ± 0.094^a^	3.811 ± 0.101^b^	3.812 ± 0.107^b^	*p* < 0.001	0.784	0.007
Ct.Ar/Tt.Ar (%)	24.8 ± 0.8^c^	32.7 ± 0.6^b^	34.9 ± 1.0^a^	30.7 ± 0.8*^c^	36.0 ± 1.1*^b^	39.6 ± 1.2*^a^	*p* < 0.001	p < 0.001	0.165
Ct.Th (mm)	0.088 ± 0.004^c^	0.139 ± 0.002^b^	0.150 ± 0.004^a^	0.109 ± 0.004*^c^	0.152 ± 0.005*^b^	0.169 ± 0.007*^a^	*p* < 0.001	0.003	0.162
Ps.Pm (mm)	8.578 ± 0.072^a^	8.174 ± 0.116^b^	8.292 ± 0.121^b^	8.397 ± 0.116^a^	8.165 ± 0.148^b^	8.311 ± 0.133^ab^	*p* < 0.001	0.044	0.095
Ec.Pm (mm)	7.125 ± 0.099^a^	6.349 ± 0.114^b^	6.406 ± 0.127^b^	6.760 ± 0.124^a^	6.198 ± 0.088^b^	6.134 ± 0.152^b^	*p* < 0.001	0.110	0.189
Ma.Ar (mm^2^)	3.285 ± 0.062^a^	2.526 ± 0.082^b^	2.479 ± 0.104^b^	2.895 ± 0.067*^a^	2.439 ± 0.071^b^	2.306 ± 0.084^c^	*p* < 0.001	0.058	0.005
Ecc (no unit)	0.394 ± 0.026^b^	0.464 ± 0.020^ab^	0.475 ± 0.014^a^	0.388 ± 0.017^c^	0.436 ± 0.031^b^	0.451 ± 0.037^a^	0.026	0.505	0.852

Different letters in a row denote statistical significance (*p* < 0.05) within a group over time by repeated measures ANOVA

Data are expressed as mean ± SEM, *n* = 10 (CON group), *n* = 8 (HSP + NAR group)

*BV* bone volume, *BV/TV* bone volume fraction, *Conn.D* connectivity density, *Ct.Ar* cortical bone area, *Ct.Ar/Tt.Ar* cortical area fraction, *Ct.Th* cortical thickness, *DA* degree of anisotropy, *Ecc* mean eccentricity, *Ec.Pm* endocortical perimeter, *Ma.Ar* marrow area, *Ps.Pm* periosteal perimeter, *Tb.Th* trabecular thickness, *Tb.N* trabecular number, *Tb.Sp* trabecular separation, *TMD* tissue mineral density, *Tt.Ar* total cross-sectional area inside the periosteal envelope, *TV* total volume

* Significantly different (*p* < 0.05) compared to CON within the same month

For cortical bone at the proximal tibia, a significant two-way interaction between age and diet was observed for Tt.Ar (*p* = 0.007), and Ma.Ar (*p* = 0.005) (Table 1). Simple main effects conducted to differentiate differences between CON and HSP + NAR groups demonstrated that Ma.Ar was lower (*p* = 0.001) in the HSP + NAR group versus CON at 2 months of age and not at other time points. Simple main effects between groups were not observed for Tt.Ar at any age. In addition to interactions between age and diet, a main effect of diet was observed for Ct.Ar (*p* = 0.014), Ct.Ar/Tt.Ar (*p* < 0.001), and Ct.Th (*p* = 0.003) whereby maternal consumption of HSP + NAR resulted in greater Ct.Ar (*p* < 0.05), Ct.Ar/Tt.Ar (*p* < 0.05), and Ct.Th (*p* < 0.05) compared to CON at all time points (Table 1, Fig. 2). A diet effect was also observed for Ps.Pm (*p* = 0.044); however, simple effects were not observed between CON and HSP + NAR groups at any age. A main effect of age was observed for all cortical parameters at the proximal tibia (*p* < 0.05) whereby increases from 2 to 6 months of age were observed for TMD, Ct.Ar, Ct.Ar/Tt.Ar, Ct.Th, and ECC in both CON and HSP + NAR groups while both groups experienced decreases in Tt.Ar, Ps.Pm Ec.Pm, and Ma.Ar from 2 to 4 months of age that persisted to 6 months of age (Table 1, Fig. 2).

At the tibia midpoint, there was a significant interaction between age and diet for Ma.Ar (*p* = 0.026) and Ec.Pm (*p* = 0.016); however, there were no simple main effects between CON and HSP + NAR groups (*p* > 0.05) at any age (Table 2). A main effect of diet was observed for TMD (*p* = 0.020) and Ct.Th (*p* = 0.044) whereby HSP + NAR offspring experienced greater TMD (*p* = 0.001) and Ct.Th (*p* = 0.009) compared to CON offspring at the tibia midpoint at 2 months of age. At 4 and 6 months of age, these differences in TMD and Ct.Th were no longer present between groups (*p* > 0.05). A significant main effect of age was observed for all cortical structural outcome measures (*p* < 0.01) with the exception of Ecc (*p* = 0.125) whereby increases in TMD, Ct.Ar, Tt.Ar, Ct.Ar/Tt.Ar, Ct.Th, and Ps.Pm were observed at the tibia midpoint of both CON and HSP + NAR offspring from 2 to 6 months of age. These changes were accompanied by significant decreases in Ec.Pm and Ma.Ar in both groups from 2 to 6 months of age (Table 2).

**Table 2:** Cortical tissue mineral density (TMD) and bone structure of the right midpoint tibias of male CD-1 offspring from mothers that were fed a control (CON) or 0.5% hesperidin (HSP) + 0.25% naringin (NAR) diet throughout pregnancy and lactation

Outcomes	CON			HSP + NAR			*p* value		
	Age (months)								
	2	4	6	2	4	6	Age	Diet	Age × diet
Cortical structure									
TMD (g/cm^3^)	1.043 ± 0.003^c^	1.135 ± 0.006^b^	1.168 ± 0.005^a^	1.070 ± 0.006*^c^	1.145 ± 0.008^b^	1.184 ± 0.008^a^	*p* < 0.001	0.020	0.144
Ct.Ar (mm^2^)	0.829 ± 0.039^c^	0.977 ± 0.041^b^	1.080 ± 0.039^a^	0.879 ± 0.015^b^	1.091 ± 0.045^a^	1.121 ± 0.045^a^	*p* < 0.001	0.186	0.131
Tt.Ar (mm^2^)	1.469 ± 0.072^b^	1.538 ± 0.079^ab^	1.640 ± 0.073^a^	1.450 ± 0.028^b^	1.661 ± 0.041^a^	1.642 ± 0.047^a^	*p* < 0.001	0.661	0.062
Ct.Ar/Tt.Ar (%)	56.2 ± 0.6^c^	63.9 ± 0.9^b^	66.2 ± 1.0^a^	60.6 ± 1.2^c^	65.6 ± 1.4^b^	68.2 ± 1.0^a^	*p* < 0.001	0.066	0.130
Ct.Th (mm)	0.200 ± 0.005^c^	0.239 ± 0.004^b^	0.256 ± 0.005^a^	0.220 ± 0.005*^c^	0.255 ± 0.010^b^	0.273 ± 0.009^a^	*p* < 0.001	0.044	0.910
Ps.Pm (mm)	4.872 ± 0.146^b^	5.096 ± 0.179^ab^	5.336 ± 0.157^a^	4.851 ± 0.065^b^	5.403 ± 0.100^a^	5.241 ± 0.100^a^	*p* < 0.001	0.718	0.069
Ec.Pm (mm)	3.384 ± 0.129^a^	3.100 ± 0.143^b^	3.092 ± 0.136^b^	3.154 ± 0.051^a^	3.163 ± 0.069^a^	2.956 ± 0.048^b^	*p* < 0.001	0.510	0.016
Ma.Ar (mm^2^)	0.640 ± 0.035^a^	0.560 ± 0.041^b^	0.559 ± 0.038^b^	0.574 ± 0.026^ab^	0.570 ± 0.021^a^	0.520 ± 0.014^b^	0.001	0.468	0.026
Ecc (no unit)	0.705 ± 0.010	0.711 ± 0.017	0.704 ± 0.013	0.712 ± 0.013	0.732 ± 0.011	0.712 ± 0.013	0.125	0.502	0.541

Different letters in a row denote statistical significance (*p* < 0.05) within a group over time by repeated measures ANOVA

Data are expressed as mean ± SEM, *n* = 10 (CON group), *n* = 8 (HSP + NAR group)

*Ct.Ar* cortical bone area, *Ct.Ar/Tt.Ar* cortical area fraction, *Ct.Th* cortical thickness, *Ecc* mean eccentricity, *Ec.Pm* endocortical perimeter, *Ma.Ar* marrow area, *Ps.Pm* periosteal perimeter, *Tt.Ar* total cross-sectional area inside the periosteal envelope

* Significantly different (*p* < 0.05) compared to CON within the same month

### Ex Vivo BMD, Trabecular, and Cortical Bone Structure at the Femur and Lumbar Vertebrae

**Fig. 3:**
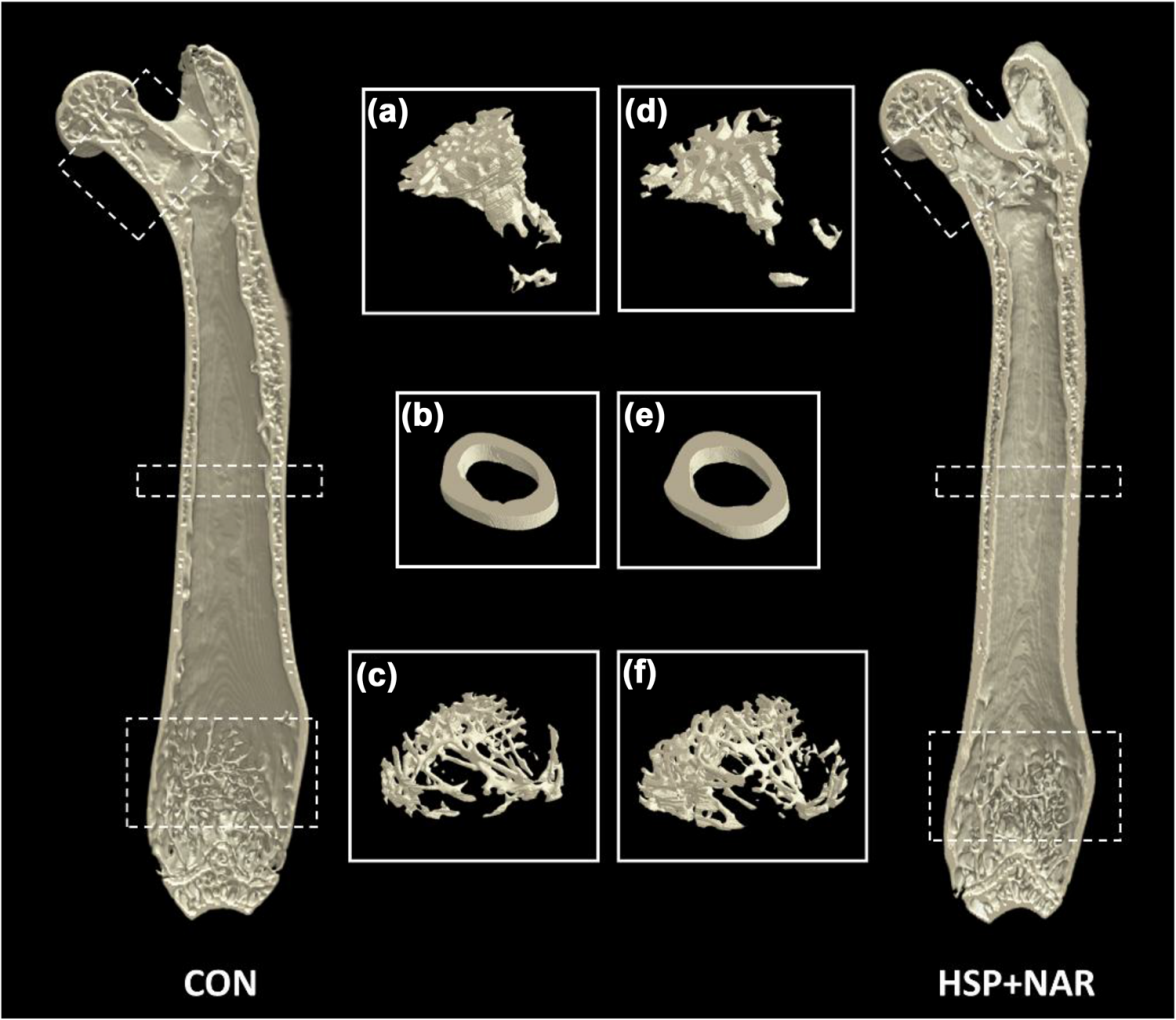
Representative images of trabecular and cortical bone structure at the femur (coronal view) of male CD-1 offspring at 6 months of age from mothers that were fed 0.5% hesperidin (HSP) combined with 0.25% naringin (NAR) or the control (CON) diet throughout pregnancy and lactation. Femurs of offspring were scanned ex vivo: **a**, **d** trabecular bone at the femur neck, **b**, **e** cortical bone at the femur midpoint, and **c**, **f** trabecular bone at the distal femur of CON and HSP + NAR groups. The regions of interest are also depicted within the dashed boxes

At the femur, no differences in BMD (*p* = 0.540), bone mineral content (BMC) (*p* = 0.997), and bone area (*p* = 0.287) were observed between CON and HSP + NAR groups (Table 3). At the femur neck, maternal consumption of HSP + NAR resulted in lower Tb.Sp (*p* = 0.049) in male offspring while no differences in other trabecular structural properties were observed (*p* > 0.05) (Table 3, Fig. 3). There were no differences in cortical structure at the femur midpoint (*p* > 0.05) or trabecular structure at the distal femur (*p* > 0.05) between CON and HSP + NAR groups (Table 3, Fig. 3).

**Table 3:** Whole femur bone mineral density (BMD), bone mineral content (BMC), area, and bone structure at the neck, midpoint, and distal regions of the femur of male CD-1 mice from mothers that were fed a control (CON) or 0.5% hesperidin (HSP) + 0.25% naringin (NAR) diet throughout pregnancy and lactation

	CON	HSP + NAR	*p* value
Femur BMD (g/cm^2^)	0.089 ± 0.003	0.091 ± 0.003	0.540
Femur BMC (g)	0.044 ± 0.002	0.044 ± 0.002	0.997
Femur Area (cm^2^)	0.497 ± 0.011	0.482 ± 0.008	0.287
Femur neck trabecular structure			
TV (mm^3^)	0.540 ± 0.046	0.504 ± 0.032	0.533
BV (mm^3^)	0.205 ± 0.020	0.211 ± 0.010	0.782
BV/TV (%)	37.9 ± 1.7	42.7 ± 2.5	0.128
Tb.Th (mm)	0.098 ± 0.002	0.102 ± 0.004	0.453
Tb.N (1/mm)	3.854 ± 0.158	4.190 ± 0.175	0.176
Tb.Sp (mm)	0.189 ± 0.009	0.166 ± 0.006*	0.049
Femur midpoint cortical structure			
Ct.Ar (mm^2^)	1.401 ± 0.055	1.486 ± 0.082	0.408
Tt.Ar (mm^2^)	2.823 ± 0.139	2.736 ± 0.093	0.087
Ct.Ar/Tt.Ar (%)	50.0 ± 1.6	54.2 ± 5.3	0.112
Ct.Th (mm)	0.245 ± 0.008	0.267 ± 0.011	0.119
Distal femur trabecular structure			
BV/TV (%)	11.5 ± 1.2	13.4 ± 0.981	0.232
Tb.Th (mm)	0.073 ± 0.001	0.076 ± 0.002	0.203
Tb.N (1/mm)	1.594 ± 0.166	1.778 ± 0.139	0.411
Tb.Sp (mm)	0.381 ± 0.050	0.339 ± 0.027	0.465

Data are expressed as mean ± SEM, *n* = 8/group

*BV* bone volume, *BV/TV* bone volume fraction, *Ct.Ar* cortical bone area, *Ct.Ar/Tt.Ar* cortical area fraction, *Ct.Th* cortical thickness, *Tb.Th* trabecular thickness, *Tb.N* trabecular number, *Tb.Sp* trabecular separation bone, *Tt.Ar* total cross-sectional area inside the periosteal envelope, *TV* total volume

* Significantly different compared to CON group by unpaired, two-tailed Student’s *t* test

**Fig. 4:**
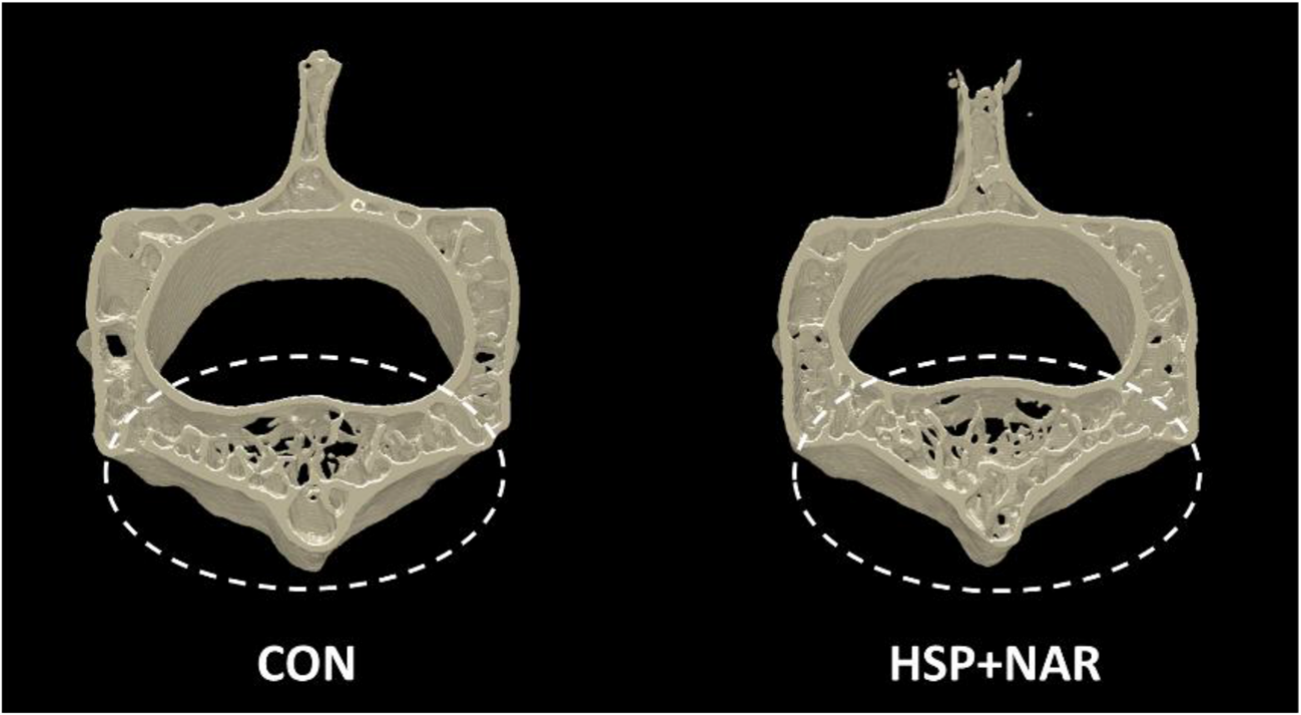
Representative images of trabecular and cortical bone structure at the second lumbar vertebra (LV2) (transverse view) of male CD-1 offspring at 6 months of age from mothers that were fed 0.5% hesperidin (HSP) combined with 0.25% naringin (NAR) or the control (CON) diet throughout pregnancy and lactation. LV2 were scanned ex vivo. The region of interest included the cortical and trabecular bone in the vertebral body and is depicted within the dashed circle

At LV1-3, no differences in BMD (*p* = 0.500), BMC (*p* = 0.370), and bone area (*p* = 0.630) were observed between CON and HSP + NAR groups (Table 4). However, higher TV (*p* = 0.002) and BV (*p* = 0.016) were observed in trabecular bone at LV2 in HSP + NAR group compared to CON (Table 4, Fig. 4). No other differences (*p* > 0.05) in trabecular or cortical bone structure at LV2 were observed between CON and HSP + NAR groups (Table 4, Fig. 4).

**Table 4:** LV1-3 bone mineral density (BMD), bone mineral content (BMC), area, and bone structure at the vertebral body of male CD-1 mice from mothers that were fed a control (CON) or 0.5% hesperidin (HSP) + 0.25% naringin (NAR) diet throughout pregnancy and lactation

	CON	HSP + NAR	*p* value
LV1-3 BMD (g/cm^2^)	0.077 ± 0.002	0.079 ± 0.002	0.500
LV1-3 BMC (g)	0.032 ± 0.001	0.034 ± 0.001	0.370
LV1-3 Area (cm^2^)	0.413 ± 0.013	0.422 ± 0.013	0.630
LV2 trabecular structure			
TV (mm^3^)	1.071 ± 0.081	1.435 ± 0.035*	0.002
BV (mm^3^)	0.250 ± 0.024	0.364 ± 0.035*	0.016
BV/TV (%)	22.9 ± 1.04	26.0 ± 2.2	0.337
Tb.Th (mm)	0.070 ± 0.001	0.072 ± 0.001	0.331
Tb.N (1/mm)	3.270 ± 0.138	3.512 ± 0.262	0.397
Tb.Sp (mm)	0.235 ± 0.008	0.232 ± 0.012	0.978
LV2 cortical structure			
Ct.Ar (mm^2^)	0.268 ± 0.008	0.266 ± 0.017	0.918
Ct.Th (mm)	0.086 ± 0.003	0.081 ± 0.004	0.290

*BV* bone volume, *BV/TV* bone volume fraction, *Ct.Ar* cortical bone area, *Ct.Th* cortical thickness, *Tb.Th* trabecular thickness, *Tb.N* trabecular number, *Tb.Sp* trabecular separation, *TV* total volume

* Significantly different compared to CON group unpaired, two-tailed Student’s *t* test. Data are expressed as mean ± standard error of the mean (SEM), *n* = 10 (CON group), *n* = 7 (HSP + NAR group)

### Biomechanical Strength at the Tibia and Femur Midpoint and at the Femur Neck

There were no differences in peak load at the tibia midpoint (CON 21.9 ± 0.9 N; HSP + NAR 22.1 ± 1.8 N, *p* = 0.934), femur midpoint (CON 31.5 ± 1.9 N; HSP + NAR 33.2 ± 1.7 N, *p* = 0.517), or femur neck (CON 28.6 ± 0.7 N; HSP + NAR 27.6 ± 1.8 N, *p* = 0.607).

## Discussion

Maternal consumption of HSP + NAR during pregnancy and lactation resulted in nutritional programming such that cortical and trabecular bone structures were improved in male CD-1 offspring. Moreover, the programming effects of HSP + NAR were sustained in cortical bone at the proximal tibia. Specifically, cortical structure at the proximal tibia was greater at 2, 4, and 6 months of age in offspring of mothers fed HSP + NAR compared to offspring of CON mothers, while benefits to cortical bone structure and cortical TMD at the midpoint tibia and trabecular bone structure at the proximal tibia did not persist to later adulthood and did not translate into stronger bones at 6 months of age. At other skeletal sites, offspring from HSP + NAR mothers experienced small but significant improvements in trabecular bone structure at the femur neck and LV2 at 6 months of age, further demonstrating the nutritional programming effects of HSP + NAR on bone structure in male CD-1 offspring.

In contrast to our previous findings in female CD-1 offspring [7], data from the present study in male siblings do not support our hypothesis that maternal consumption of HSP and NAR during pregnancy and lactation compromises the development of bone mineral and bone structure. In female offspring, maternal exposure to HSP + NAR resulted in a 27–38% lower BV/TV at the proximal tibia compared to CON offspring from 2 to 6 months of age [7]. These observations are in contrast to the present study which demonstrated a 5–55% higher BV/TV in male offspring whose mothers were fed HSP + NAR. Significant and sustained benefits to cortical bone at the proximal tibia were also observed in male HSP + NAR offspring including a 10–24% higher Ct.Ar/Tt.Ar compared to CON offspring over the study duration. These effects on cortical bone at the proximal tibia were not exhibited in the female HSP + NAR offspring.

While long-lasting benefits to the proximal tibia cortical bone structure were observed in HSP + NAR mice, benefits to midpoint tibia cortical bone structure and proximal tibia trabecular bone structure did not persist past 2 and 4 months of age, respectively. Thus, responses to HSP + NAR at the tibia are site-specific and are longest lasting with regards to cortical bone structure at the proximal tibia. At the femur, lower Tb.Sp was observed in the femur neck of HSP + NAR offspring; however, trabecular bone structure at the distal femur, cortical bone structure at the midpoint, and whole BMD did not differ between both groups at 6 months of age. The absence of long-term benefits to whole BMD and structure at the midpoint of the tibias and femurs likely explain why maternal exposure to HSP + NAR did not result in a higher peak load at these sites. At the LV, higher BV and TV was observed in HSP + NAR offspring while cortical bone structure and BMD did not differ between both groups. The reasons to explain these site-specific responses to HSP + NAR are unclear and further study should be undertaken to identify whether they may be related to the interactions between treatment and site-specific sensitivities to mechanical forces and developmental changes.

Doses of 0.5% HSP and 0.25% NAR that were selected for the present study represent doses that were effective in protecting against the loss of BMD, biomechanical strength, and/or deterioration of bone tissue in rodent models of aging and osteoporosis [14, 15, 20, 29–31]. In humans, 0.5% HSP and 0.25% NAR doses translate to consumptions between 400 mL and 1 L of orange or grapefruit juices [11, 32]. Thus, 0.5% HSP and 0.25% NAR represent doses that provide a proof of efficacy for these flavanone glycosides in a model of nutritional programming on bone development and not for consideration of making recommendations on the consumptions of orange and grapefruit juices.

Findings of the present study are limited in that potential mechanisms of action were not elucidated, including whether the observed effects may be attributed to HSP, NAR, or their combination and if it is exposure in utero and/or during suckling that has long-lasting effects on bone structure. To study the timing of exposure, offspring exposed to HSP + NAR could be cross-fostered at birth to mothers who have been continuously fed control diet. Given it is unknown whether HSP and/or NAR metabolites directly modulate skeletal development in fetal or suckling offspring or whether the physiological effects to the mothers indirectly alter the skeleton of offspring, epigenetic mechanisms could be investigated in both mothers and offspring [33]. Thus, future studies are needed to determine if maternal transmission of HSP and NAR metabolites to offspring occur in utero and/or through mother’s milk. Such studies could include measurement of HSP and NAR metabolites in cord blood of neonates [34] and in mother’s milk [35, 36]. Moreover, the mechanisms to explain the sex-specific effects of maternal consumption of HSP + NAR on bone mineral and bone structure in male and female CD-1 offspring require investigation.

In conclusion, while our previous study demonstrated that maternal consumption of 0.5% HSP combined with 0.25% NAR during pregnancy and lactation resulted in detriments to BMD and bone structure at the proximal tibia at 2 and 4 months of age in female CD-1 offspring [7], long-lasting benefits to bone structure in male sibling CD-1 offspring suggest a sex-specific response. Mechanisms to explain sex-specific responses require future investigation. Moreover, that the benefits to tibia structure with maternal exposure to HSP + NAR were most robust at 2 and 4 months of age points to a window of opportunity when skeletal development of male offspring may be further supported through diet. Identification of a mechanism of action may suggest other early life dietary strategies that may benefit bone structure.
